# Naive and memory T cells TCR–HLA-binding prediction

**DOI:** 10.1093/oxfimm/iqac001

**Published:** 2022-05-26

**Authors:** Neta Glazer, Ofek Akerman, Yoram Louzoun

**Affiliations:** Department of Mathematics, Bar-Ilan University, Ramat Gan, Israel; Department of Mathematics, Bar-Ilan University, Ramat Gan, Israel; Department of Mathematics, Bar-Ilan University, Ramat Gan, Israel

**Keywords:** TCR, receptor, HLA, MHC, binding prediction, memory T-cell, machine learning, two stages

## Abstract

T cells recognize antigens through the interaction of their T cell receptor (TCR) with a peptide-major histocompatibility complex (pMHC) molecule. Following thymic-positive selection, TCRs in peripheral naive T cells are expected to bind MHC alleles of the host. Peripheral clonal selection is expected to further increase the frequency of antigen-specific TCRs that bind to the host MHC alleles. To check for a systematic preference for MHC-binding T cells in TCR repertoires, we developed Natural Language Processing-based methods to predict TCR**–**MHC binding independently of the peptide presented for Class I MHC alleles. We trained a classifier on published TCR**–**pMHC binding pairs and obtained a high area under curve (AUC) of over 0.90 on the test set. However, when applied to TCR repertoires, the accuracy of the classifier dropped. We thus developed a two-stage prediction model, based on large-scale naive and memory TCR repertoires, denoted T**C**R H**LA**-b**i**nding p**re**dictor (CLAIRE). Since each host carries multiple human leukocyte antigen (HLA) alleles, we first computed whether a TCR on a CD8 T cell binds an MHC from any of the host Class-I HLA alleles. We then performed an iteration, where we predict the binding with the most probable allele from the first round. We show that this classifier is more precise for memory than for naïve cells. Moreover, it can be transferred between datasets. Finally, we developed a CD4–CD8 T cell classifier to apply CLAIRE to unsorted bulk sequencing datasets and showed a high AUC of 0.96 and 0.90 on large datasets. CLAIRE is available through a GitHub at: https://github.com/louzounlab/CLAIRE, and as a server at: https://claire.math.biu.ac.il/Home.

## INTRODUCTION

T lymphocytes (T cells) play a pivotal role in the adaptive immune response [1, 2]. T cells recognize (T cell receptor [TCR]) antigenic peptides bound to major histocompatibility complexes (MHCs) through their TCR [3, 4]. CD4 T cells typically bind MHC-II-bound peptides, while CD8 T cells bind MHC-I-bound peptides [5]. The TCR is composed of a *β* chain and an *α* chain. In the TCR *β* chain, the complementarity-determining region (CDR) 1 and CDR2 loops of the TCR contact the MHC α-helices, while the hypervariable CDR3 regions interact mainly with the peptide [1, 2], but also with the MHC [6–8]. In both TCR*α* and TCR*β* chains, CDR3 loops have the highest sequence diversity and are the principal determinants of peptide-binding specificity [9]. The CDR1 and CDR2 are fully determined by the *V* gene of the TCR, while the CDR3 is determined by *V* (*D* in the TCR*β*) and *J*, and by the addition and removal of nucleotides at the VD and DJ junctions (VJ in the *α* chain) [1, 10].

Over the last decade, large-scale bulk repertoire sequencing methods have been developed [11]. The sequenced peripheral T cells have reached the periphery. We thus assume that they were positively selected in the thymus for weak binding to peptide-major histocompatibility complex (pMHC) complexes [12]. Similarly, we assume that antigen-specific T cells are further selected based on strong pMHC binding [13]. We thus suggest that sequenced TCRs from large-scale Bulk Sequencing Experiments (BSEs) of naive T cells contain mainly TCRs binding to at least one of the host MHC alleles, and antigen-specific T cells even more so. Specifically, one may propose that in general memory T cells bind with higher affinity to the host MHC alleles than naive T cells. We here show that TCRs are indeed associated with the host MHC alleles, and this association can be used to predict TCR**–**MHC binding.

Human MHC alleles are denoted as human leukocyte antigen (HLA). The HLA genes can be classified into MHC Classes I and II. Class I MHC (A, B and C) presents intra-cellular peptides to CD8 T cells and is ubiquitously expressed on all nucleated cells [5]. Class II MHC molecules (DQ and DR) present extra-cellular antigens to CD4 T cells [14] and are only expressed on antigen-presenting cells, such as B cells, macrophages and dendritic cells [5, 14].

**Figure 1:**
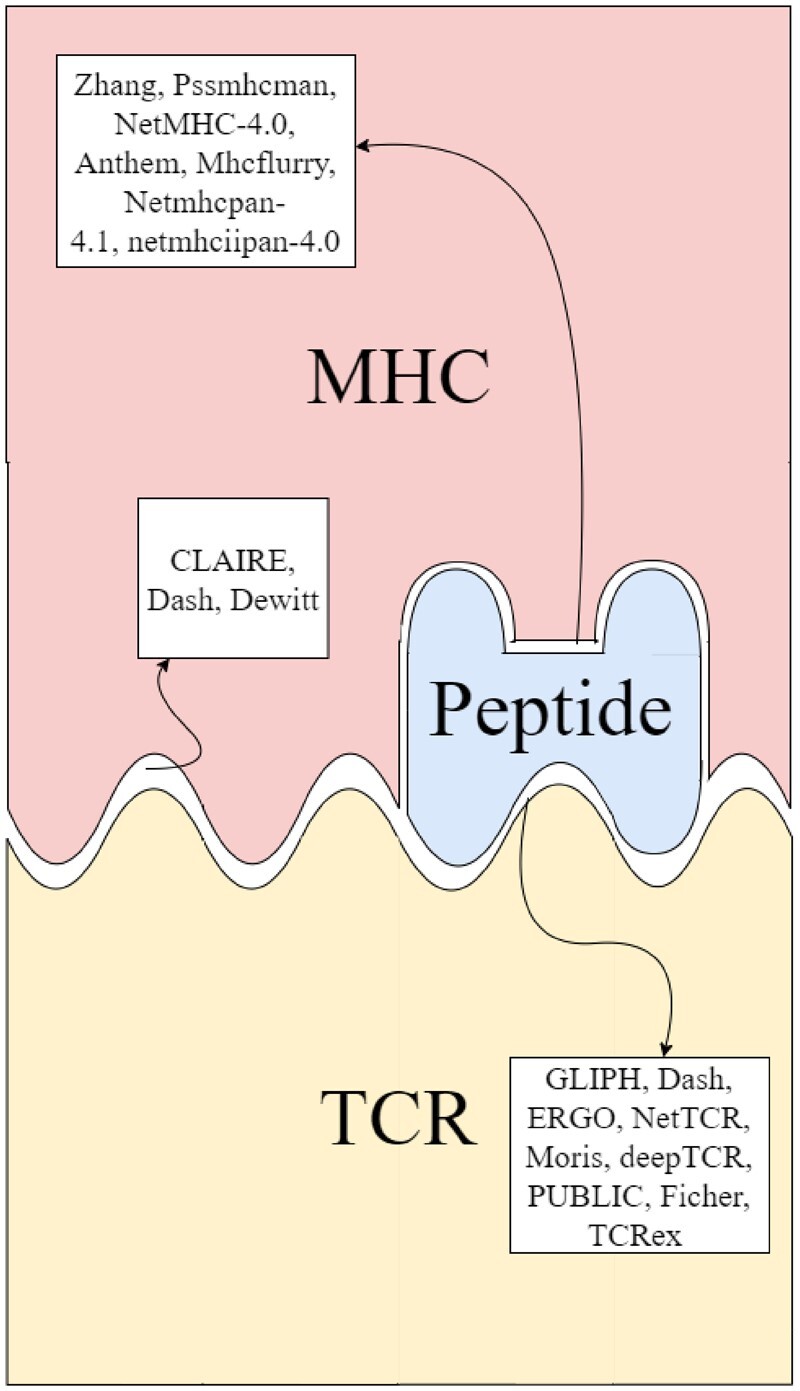
Models for the prediction of bindings between different entities in the TCR–MHC–peptide complex: MHC–peptide: The model by [15], Pssmhcman [16], NetMHC-4.0 [17], Anthem [18], MHCflurry [19], Netmhcpan-4.1 and netmhciipan-4.0 [20]. TCR–peptide: GLIPH [4], the model by [27], ERGO [28], NetTCR’s [29, 30] model, deepTCR [31], PUBLIC [32], the models presented by [33] and TCRex [34]. TCR–MHC: CLAIRE (our paper). Dash et al. [27] and DeWitt et al. [51] presented a correlation between TCR clusters and HLA association.

Multiple computational tools were developed to study and predict different components of the TCR**–**pMHC binding (Fig. 1). Excellent solutions for MHC-peptide-binding affinity prediction were proposed (especially for Class I) using both data analysis methods [15, 16] and machine learning (ML) methods [17–25] (for an excellent review, see [26]). Recently, two new layers were added to these predictions. ML and data analysis algorithms for the prediction of TCR–peptide binding, both on single TCR**–**peptide binding [4, 27–40] and on immune repertoire antigen binding [31, 38, 41–46] were developed. These algorithms were mainly based on the CDR3 sequences of the *β* chain, but some also used information about the Vβ and Jβ genes as well as the *α* chain. Recently, advanced ML models have been constructed that improved the quality of TCR**–**peptide-binding prediction drastically. ERGO2 [35] and NetTCR2.0 [36] used convolutional neural networks (CNNs) and auto-encoder projections, respectively, to encode the TCRs and the peptide. Further learning on these projections results in accurate binding predictions.

In parallel, algorithms were developed to predict MHC-binding peptides that can induce an immune response [47, 48], since not all peptides presented on the MHC molecule can actually induce a T cell response. However, there remains one last part of the interaction that has hardly been studied—the TCR**–**MHC binding. There are currently very limited models for TCR**–**HLA association (see ‘Related work’ section), and a no ML-based TCR**–**MHC-binding prediction algorithm. We here propose to develop such an algorithm for A and B HLA alleles and test the algorithm accuracy on naive and memory TCR repertoires.

## RELATED WORK

Recent studies presented a relation between Vβ and Vα gene usage and *MHC* genes. Specifically, Sharon *et al*. [49] used Bayesian inference to prove that amino acid residues encoded in the MHC molecules affect the expression of TCR Vα genes. Johnson *et al*. [50] detected a correlation between increased HLA allele sharing and an increased number of shared TCR*β* clones. Dash *et al*. [27] used TCR distance-based clustering and demonstrated a connection between TCR similarity and HLA allele association. Motif-based clustering has been shown to also create groups connected by the binding ability to the same HLA [4]. DeWitt *et al*. [51] have used a combination of TCRdist developed by Dash *et al*. [27] and statistical analysis of co-occurrence patterns in cohorts and clustered TCR sequences. They have also presented a connection between their clustering and HLA association. Moreover, Dash *et al*. [27] have also shown a correlation between the charge in a specific position in the HLA-loci, and the charge of the CDR3 sequence. However, none of them developed a specific predictor of TCR**–**MHC binding. We here propose a novel method to develop such a binding prediction algorithm.

## METHODS

### Datasets

To predict whether a given TCR (*t_k_*) binds a given HLA (*h_i_*), we used two types of datasets (Table 1):

**Table 1:** Dataset information for all the datasets used in the article

	McPAS	VDJdb	Miron	Emerson	Sallusto	Gold	van Heijst
Clones	12 000	40 000	861 172	218 747 000	2 797 756	39 735 921	300 000
Samples	NA	NA	132	626	49	294	56
Memory T cells	Yes	No	Yes	No	No	No	No
Type	PTH	PTH	BSE	BSE	BSE	BSE	BSE

The clones column is the total number of distinct TCRs in the data. The sample number is the number of different samples in the dataset, some from the same subject. Some datasets are only of memory T cells (McPAS, Miron), while others contain also nonmemory T cells as well.

**Table 2:** Notations Table

Notation	Definition
*h _i_*	The *i*-th HLA gene
*v_j_*	The *j*-th Vβ-gene
*t_k_*	The *k*-th TCR
*d_l_*	The *l*-th donor
*H_l_*	The set of HLA genes present in donor *d_l_*
*H_k_*	The set of HLA genes present in the donor from which *t_k_* was sampled
*E_t_*	The real vector encoding of the TCR
*E_h_*	The real vector encoding of the HLA
*E_th_*	The concatenated real vector encoding of the TCR and HLA
$s_{i,j}$	$P(h_{i}|v_{j})$ : The probability that a TCR binds to HLA *h_i_* given that its Vβ-gene is *v_j_*. Calculated by the Naive Bias model
$Z_{k,i}$	$P(h_{i}|t_{k})$ : The real probability that TCR *t_k_* binds to HLA *h_i_*. Can be either categorical or probabilistic

Notations table. All of the notations and their definitions used in the article are provided in the table.

- **paired TCR–HLA (PTH)**: Datasets with PTH (*t_k_*, *h_i_*), where a specific TCR $\left(t_{k}\right)$ is known to bind a peptide in the context of a given HLA allele $\left(h_{i}\right)$. In this analysis, we ignored the peptide, and only focused on the HLA. We used the following datasets:
  - McPAS [52] is a manually curated database of TCR sequences and their associated MHC, based on published literature, with more than 20 000 TCR*β* sequences;
- VDJdb [53] is an open, comprehensive database of over 40 000 TCR sequences and over 200 cognate epitopes and the restricting MHC allotype acquired by manual processing of published studies.
- **BSE**: Sets of TCRs from the repertoire sequencing of a specific compartment (or whole blood) from a given donor (*d_l_*). We used datasets where each donor is associated with a set of HLA A and B alleles ($H_{l}=\{h_{l_{1}},h_{l_{2}},h_{l_{3}},h_{l_{4}}\}$). We ignored the C alleles since information on C was very limited. We assume that each TCR in this donor binds at least weakly to one of the host HLAs. We used four datasets as follows (see also Table 1).
  - The Miron dataset contains memory T cells from 11 donors, 9 of them with HLA typing [54].
  - The Sallusto dataset [55].
  - The Gold dataset contains TCR repertoire longitudinally throughout pregnancy [56].
  - The Van Heijst dataset [57] contains TCRs from 27 patients, at either 6 or 12 months after stem cell transplants or double-unit umbilical cord blood transplants [58].
  - The Emerson dataset contains T cell repertoires of 666 subjects with known cytomegalovirus serostatus by immunosequencing, and an independent validation cohort of 120 subjects [41]. The Emerson dataset was taken from https://clients.adaptivebiotech.com/pub/emerson-2017-natgen. It is the only dataset used here that was not separated into CD4 and CD8 T cells.

### TCR pre-processing

#### McPAS and VDJdb preprocessing

For some peptides in the McPAS dataset, MHC information is also available. To use McPAS for TCR**–**HLA predictions, we filtered out the following TCRs:

- non-human TCRs;
- TCRs with missing information about the HLA allele;
- TCRs with two or more HLA alleles; and
- TCRs with missing CDR3*β* sequence.

We translated HLA to a four digits HLA representation (e.g. HLA A*02:01), and Vβ gene, and Jβ-gene representations to two fields Vβ-gene number (e.g. V01−02), and one field Jβ-gene family (e.g. *J*02). When only two-digit HLA information was available, we used the most frequent HLA allele in the group (e.g. HLA−A*02→HLA−A*02:01, V05→V05−01). Moreover, all single-field Vβ genes in the dataset were expanded to a two-field representation by replacing them with the most frequent allele in the same group. Finally, different alleles of the same Vβ gene were ignored (V01-02:01 → V01-02), and when multiple options for a Vβ gene were present in the dataset, the first one was chosen (V13−01/3/4→V13−01).

Beyond that, VDJdb was filtered as in ERGO [28].

### Naive Bayes Vβ-gene-based MHC-binding prediction

To predict the association between CDR1 and CDR2 regions of a TCR and an HLA allele, we built a Naive Bayes classifier for the different HLA alleles. Note by $h_{1},h_{2},..,h_{N}\in H$, the group of all HLA alleles in a dataset, and $v_{1},v_{2},\ldots ,v_{M}\in V$ the group of all the Vβ genes in the same dataset (See Table 2 for notation) . The following probabilities were estimated:

-
$P(h_{i})$ : The probability that a randomly chosen TCR from the dataset binds to the HLA *h_i_*.
-
$P(v_{j})$ : The probability that a random TCR from the dataset uses the Vβ-gene *v_j_*.
-
$P(v_{j}|h_{i})$ : The probability that a TCR uses the Vβ-gene *v_j_*, given that the TCR binds to HLA *h_i_*

Using these probabilities, we calculated $P(h_{i}|v_{i})$ for each HLA *h_i_* and Vβ gene *v_i_* with Equation (1).

(1) $$P(h_{i}|v_{j})=\frac{P(h_{i})P(v_{j}|h_{i})}{P(v_{j})}$$

### TCR–HLA-binding prediction neural network

The following sections are highly ML oriented, but the manuscript can be understood without them. All the terms required to understand the results are explained in the results section.

The ELATE (Encoder-based LocAl Tcr dEnsity) TCR autoencoder [59] was trained as an initialization step to the TCR**–**MHC-binding predictor. To train the ELATE TCR autoencoder, first, we represented each amino acid as a 21 dimensions one-hot vector (20 possible amino acids and an additional stop codon), where all values were set to zeros except one index of the corresponding amino acid which was set to 1. An additional position with a stop codon was added at the end of the one-hot vector. Zero padding was then added to the CDR3 vectors, completing the vectors to the maximum lengths chosen according to the data lengths distribution. The autoencoder was trained for 300 epochs only on the TCRs, and no information on the peptides or the HLA was ever used to train the autoencoder [28]. The pre-trained TCR autoencoder parameters were used as initial values for the encoder part of the TCR**–**MHC-binding prediction.

Using the encoding from the pre-training above, we developed a binary model which is trained to output 1 if the TCR and an MHC molecule bind and 0 otherwise (see Fig. 3). The model had two inputs: the TCR and the HLA allele. For the TCR, we used the CDR3*β* chain sequence, the Vβ and Jβ genes. When the *α* chain information was available, we used it too. The CDR3 amino acid chains were encoded with the TCR autoencoder. As in ERGO and ELATE [28, 59], we considered the Vβ and the Jβ genes as categorical features. To encode these features, we used a 50-dimensional embedding vector for each feature. Different embedding matrices were learned for the Vα, Vβ, Jα, Jβ genes. All the encoded T cells features were concatenated to a vector *E_t_*.

For the HLA, we used a 14-dimensional embedding vector, and a CNN encoder with two convolutional layers, each with a Relu as activation function and max-pooling, and two linear layers, with a dropout of 0.1. The output encoding (*E_h_*) had a dimension of 100. For the TCR**–**HLA-binding predictor, we used an Adam optimizer, with a learning rate of 0.007, and a weight decay of 0.001. All the T cell’s encoded features were concatenated with the HLA-encoded vector into one vector *E*_th_ that was the input of a linear layer of half the input dimension, and a sigmoid on the output of the last layer to get a probability value. The activation in the multilayer perceptron (MLP) was a Leaky ReLU. The dropout rate of 0.1 was set between layers.

### Simulated BSE dataset generation

In BSE datasets, each TCR can be associated with multiple HLA alleles. Assuming a TCR in a host was positively selected on one of the host’s HLAs, we only know which HLA alleles the host has, but not which specific HLA is associated with this specific TCR. To simulate that, we used McPAS and produced a simulated BSE by assigning each TCR with three more HLA alleles using the HLA allele distribution in McPAS.

### CD4–CD8 classification neural network

The Emerson dataset contains mixed TCR with both CD4 and CD8 T cells. We are only interested here in the binding between TCR and Class I MHC alleles and thus focused on CD8 T cells. To separate CD4 and CD8 T cells, we developed a binary classifier to identify if a TCR originates from a CD4 or a CD8 T cell. We encoded all the T cell features as mentioned in TCR**–**HLA-binding prediction section to a vector *E_t_*. The required output was set to be 1 for CD8 T cell, and 0 for CD4 T cell. The prediction was done using one hidden linear layer of half the input dimension and a ReLU activation function, followed by a single output with a sigmoid activation function. The dropout rate between layers was 0.1. We used an Adam optimizer with a learning rate of 0.0005 and a weight decay of 0.0005. Further model hyperparameters are detailed in ‘Hyperparameter optimization’ section.

We trained the model on each dataset, and then tested the trained model on all other datasets as well. When testing within a dataset, the dataset was divided into 80% training set and 20% test set. When training on one dataset and testing on another one, the entire first dataset was used as training, and the other as a test.

### Experimental setup

To create negative TCR–HLA pairs, we sampled pairs that do not exist in the positive pair dataset, while ensuring the data features are distributed equally in the positive and negative samples. In the presence of multiple donors (Miron, Emerson datasets), each donor could be either in the training or in the test, but not in both. In the model training, we used early stopping on the Area under curve (AUC) values of the internal validation, with a patience of 20 epochs.

### Hyperparameter optimization

Neural network intelligence (NNI) is a lightweight but powerful toolkit to help users automate Feature Engineering, Neural Architecture Search, Hyperparameter Tuning and Model Compression (see NNI Github: https://github.com/microsoft/nni). The parameters we optimized in both the CD4–CD8 classifier and the TCR–HLA-binding predictor are the learning rate, dropout probability and weight decay. Additional parameters that were not tuned using NNI are the embedding matrix dimension, activation functions, network size and optimizer. The hyperparameters were optimized on an internal validation set, and not on the test set.

### Two-stage model

For each TCR in a BSE, there are multiple candidate HLA alleles. This is in contrast with standard binary or multi-class classifiers, where the class of each instance is assumed to be known in the training stage. To solve that, we developed a two-stage classifier:

1. We first use multiple pairs for each input TCR**–**HLA: (*t_k_*, *h_i_*), where *t_k_* is the TCR, and *h_i_* is one of the HLAs associated with the TCR. Suppose that we have for a TCR *t_k_*, a group of possible HLAs: $H_{k}=\{h_{k_{1}},\ldots ,h_{k_{m}}\}$, the positive pairs contain all pairs: $\left(t_{k},h_{k_{1}}),\ldots ,(t_{k},h_{k_{m}}\right)$, for each TCR in the dataset. Negative pairs will contain the combinations of *t_k_* and *h_i_*, such that: $h_{i}\notin H_{k}.$
2. Train again the model on the same training dataset, but this time assigning to each TCR *t_k_*, only the pair (*t_k_*, *h_i_*) that got the highest score in the first stage above as the proper match and all others as errors.

### Evaluation

We trained the classifiers on all datasets and tested them on all the other datasets. Testing within a dataset, an 80/20 training/test sets division was used. When testing on another dataset, the entire dataset was used as training and the other as the test. We used two measurements for TCR–HLA-binding prediction performance:

1. Group accuracy (GA) is the number of correctly predicted data samples out of all the samples. Note that when we have multiple classes, we denote a correct precision if we fit any of the classes.
2. AUC—The receiver operator characteristic (ROC) curve is created by plotting the true-positive rate (TPR= TP/P) against the false-positive rate (FPR = FP/N), as a function of the threshold. TP and FP are the fraction of samples classed as positive, and are truly positive or negative, respectively; and *P* and *N* are the total number of real positive and negatives, respectively. A positive/negative classification is an above/below threshold score. The AUC is the integral of the curve producing the area under it, and is 1 for a perfect classifier and 0.5 for a random classifier [60–63].

## RESULTS

We assume that TCRs in naive and memory T cells must have bound at least weakly with a peptide in the context of a host-specific MHC (either in the thymus or both in the thymus and the periphery). We tested whether a prediction model can be developed to predict the HLA allele(s) that the TCR was bound to. However, for a given TCR in a deep sequencing experiment, one cannot know to which of the host HLA alleles it is bound in the periphery. To address this, we used two types of datasets (see dataset details in ‘Methods’ section):

- PTH datasets with paired TCR–HLA (*t_k_*, *h_i_*). In such datasets, each entry is a reported TCR–pMHC interaction. As such, we know the HLA allele to which the TCR was bound.
- BSE Sets of TCRs from a given donor (*d_l_*), where each donor is associated with a subset of HLA alleles ($H_{l}=\{h_{l_{1}},h_{l_{2}},h_{l_{3}},h_{l_{4}}\}$, since we only focus on A and B Class I alleles for which there is enough information). In such experiments, we do not know to which allele the TCR was bound, but we presume it must have been bound to one of the host HLA alleles to pass thymic (and possible peripheral) positive selection.

### PTH-based naive Bayes classifier

A simple approach can be developed if one assumes that most of the interaction between the TCR and the MHC molecule is mediated by the Vβ CDR1 and CDR2. The CDR1 and CDR2 information is fully included in the Vβ-gene allele. We thus computed the odd ratio of binding to a peptide in the context of a specific HLA allele (*h_i_*), given a known TCR(*t_k_*). We tested this ratio (formally, we trained the Naive Bayes classifier, as described in ‘Naive Bayes Vβ gene-based MHC-binding prediction’ section) on a training set of 8978 pairs of TCR–HLA pairs, and tested it on 3187 pairs from the McPAS PTH dataset. For each pair (*h_i_*, *v_j_*), we assigned a probability $s_{i,j}=P(h_{i}|v_{j})$ based on the observed pair frequencies in the training set. We then computed the same probability for each pair in the test set, leading to a score for each value in the test set. We produced random (fake) negative pairs, generated by randomly sampling TCRs and HLAs from the McPAS dataset, such that the TCR and HLA alleles distribution is the same as the real test set (for details, see ‘Evaluation’ section), and calculated the same score for the fake pairs. The quality of the prediction can be estimated using a ROC on the odd ratio. The ROC curve is summarized as a single value denoted by AUC representing the surface under the curve. An AUC value of 1 represents a perfect classification (for all real pairs, the score is above some value *T*, and for all fake ones, it is below). An AUC of 0.5 represents a random classifier that assigns similar scores to real and fake pairs.

**Figure 2:**
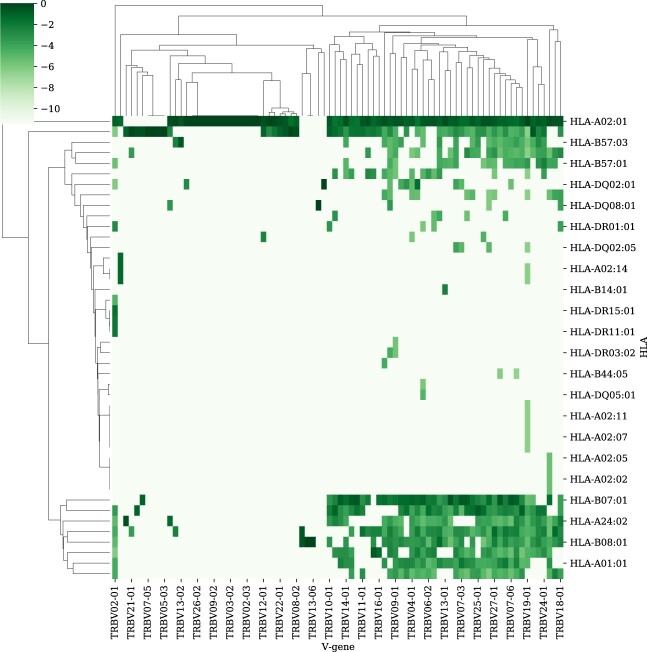
Heatmap of correlation between Vβ-genes *v_j_* and HLA alleles *h_i_*. For each *v_j_*, *h_i_* the value presented in the heatmap is $\log(P(h_{i}|v_{j})+\epsilon )$, where $\epsilon =10^{-5}$. The *V* genes and HLA alleles are clustered using single link hierarchical clustering.

The test AUC score on the odd ratio-based predictions is 0.725. Thus, one can use the TCR*β* for an initial prediction of the HLA allele that a TCR binds to. To visualize the odd ratio, we applied a two-dimensional clustering of all HLA alleles as a function of their odd ratio for each Vβ chain and of the Vβ chains as a function of their odd ratio versus each HLA allele. Interestingly, the division of the Vβ genes is not associated with the Vβ families, rather into the rare and frequent alleles, with some HLA alleles associated with the frequent Vβ genes, and other with the rare genes (see Fig. 2). Note that the Vα genes were not used for a parallel analysis, since about 94% of the TCRs in the McPAS dataset that had information on the Vα gene were bound to HLA A*02:01.

The Vβ gene-level classifier was based on a strong assumption that the CDR3 sequence does not affect the TCR–MHC interaction. However, at least a part of the CDR3 binds directly to the MHC molecule [64]. To avoid this assumption, we enlarged the classifier to include also the full CDR3 amino acid sequence.

### PTH-based CLAIRE

We used again the McPAS, and enlarged it with the VDJDB dataset, which also contains (*t_i_*, *h_i_*) pairs of TCR and pMHC pairs recognized by the TCR. We again ignored the peptide and focused only on the TCR–MHC interaction, and developed a ML model that predicts for an input pair (*t_i_*, *h_i_*) of a TCR and HLA allele whether they bind. The model is further denoted as T**C**R H**LA**-b**i**nding p**re**dictor (CLAIRE). We produced again fake pairs to match the real pairs as in the above section. The input of CLAIRE was the $V\beta ,\;J_{\beta }$ and the *β* chained CDRs sequence, as well as their parallel in the *α* chain when available.

**Figure 3:**
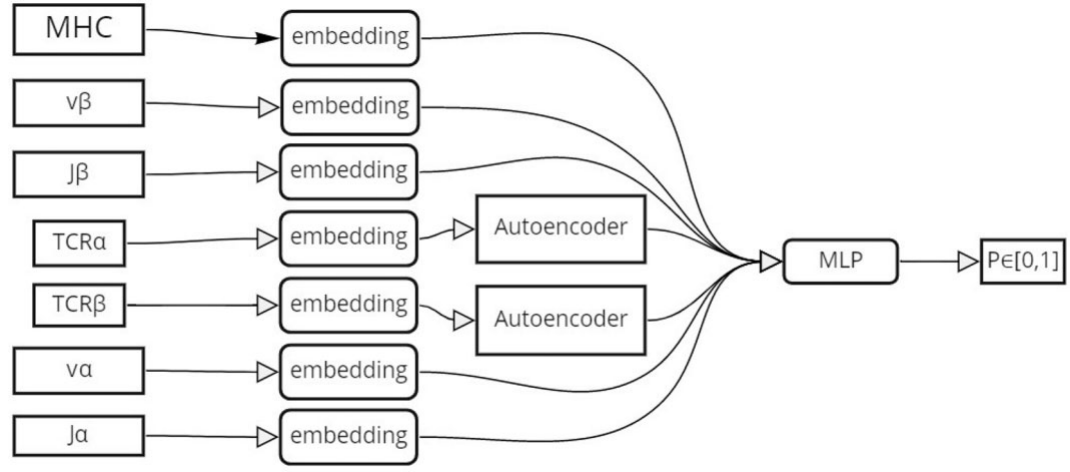
Illustration of the CLAIRE’s architecture. We developed a binary model trained to output 1 if the TCR and HLA bind and 0 otherwise. The model had TCR and HLA allele inputs. For the TCR, we used the CDR3-beta chain sequence, the Vβ and Jβ genes. When *α* chain information was available, we used it too. The CDR3 amino acid chains were encoded with a TCR Autoencoder (see ‘Methods’ section). We considered the Vβ and Jβ genes as categorical features (see ‘Methods’ section for the TCR representation). The HLA was also a categorical feature. All features were embedded as real valued vectors *E_t_* and *E_h_*, respectively. All the T cell’s encoded features were concatenated with the HLA-encoded vector into one vector *E_th_*, that was the input of a MLP. The output of the MLP is a real valued between 0 and 1 trained to be high for binding TCR–HLA pairs and low otherwise.

At the technical level, we translated (encoded) each TCR and each HLA allele separately to real-valued vectors, *E_t_* and *E_H_*. We then used these two vectors as the input to a neural network that produced a value between 0 and 1 (Fig. 3). We then optimized the projection to ensure high scores for the real pairs and low scores for the fake pairs. The technical details of the encoding and the MLP are in the ‘TCR–HLA-binding prediction neural network’ section.

**Figure 4:**
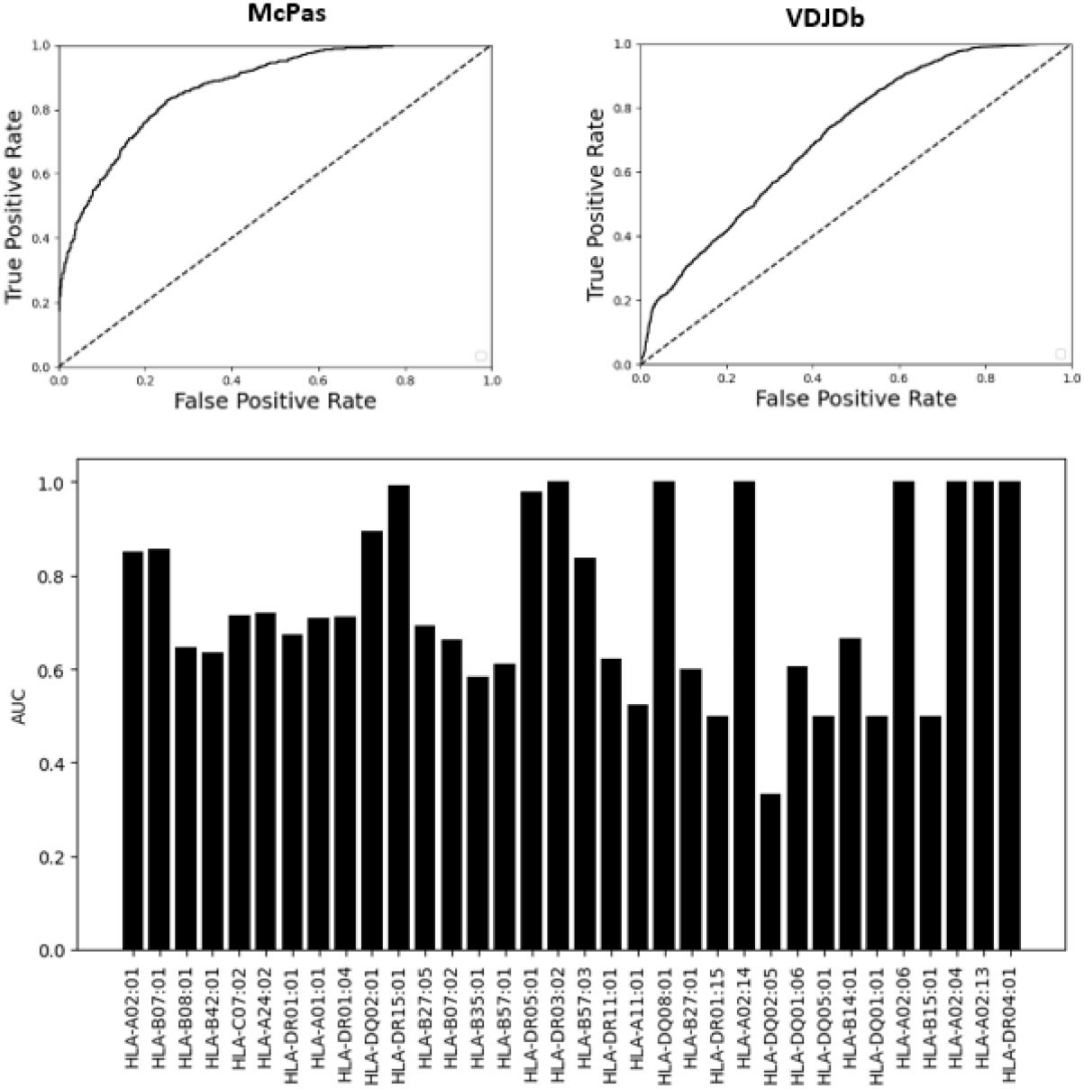
Performance of the TCR–HLA-binding model, with datasets that contain pairs of TCR and HLA (*t_k_*, *h_j_*). The upper plots are the ROC curve for the prediction based on the McPAS and VDJdb datasets. One can clearly see that the accuracy is much higher using the McPAS dataset. The bar plot contains the results of the model trained in on McPAS dataset and tested on each of the HLAs in the same dataset. This model was trained on MHC Classes I and II present in the McPAS dataset. This is the only analysis where we used samples of TCRs that bind to MHC Class II. A clear difference can be observed between HLA with very high AUC values and many other with low AUC values.

The AUC obtained from the McPAS dataset was significantly better than the one from VDJdb (AUC 0.87 versus 0.72; Fig. 4). Similar results were previously reported for the binding of TCR–peptide-binding prediction [35].

The AUC score described above was for the entire dataset, composed of multiple HLA alleles. To test whether the accuracy is uniform over all HLA allele, we computed the AUC for each HLA allele by testing the accuracy of the same classifier, on a specific HLA allele, and different TCRs either reported to bind the appropriate HLA allele, or not reported to bind it (Fig. 4). We made the conservative assumption that TCRs not reported to bind a given HLA allele do not bind it. The AUC values are highly nonuniform among HLA alleles, with no clear association between the allele frequency and the AUC, with many alleles having 0.6 AUC values, but others having very high AUC, such as HLA A*02:04 and DRB*03:02.

### Limitations of PTH models in BSE data

The McPAS-based CLAIRE classifier was trained to predict the binding of TCR and MHC based on reported experimental binding. However, such binding tends to be limited to high-affinity binding. In contrast, naïve or memory TCRs may have been selected by very weak binding. As such, the prediction obtained from the McPAS dataset may not be applicable to BSE datasets. We tested the accuracy of CLAIRE on repertoires pre-sorted into CD4 and CD8 compartments. In BSE datasets, each TCR can be associated with multiple HLA alleles. Assuming a TCR in a host was positively selected on one of the host’s HLAs, we only know which HLA alleles the host has, but not which specific HLA is associated with this specific TCR. At the mathematical level, in BSE, the classification problem can be stated as follows:

Given a set of TCRs $t_{1},t_{2},\ldots ,t_{n}$, and a set of possible HLA alleles *h*_1_, *h*_2_,…, *h_m_*, and a hidden variable $Z_{k,i}$ relating each TCR $t_{k}\in$*t*_1_, *t*_2_,…, *t_n_* to its target HLA allele $h_{i}\in$*h*_1_, *h*_2_,…, *h_m_*, so that $Z_{k,i}=P(h_{i}|t_{k})$. We only know for each $t_{k}\in$*t*_1_, *t*_2_,…, *t_n_* is associated with a subset of $H_{k}=\{h_{k_{1}},\ldots ,h_{k_{m}}\}$. This is in contrast with standard ML, where we know the specific target *h_i_* of each *t_k_*. Our goal is to predict the association of a TCR with a single HLA allele (as measured by $Z_{k,i}$). The ambiguity in the TCR target complicates the assessment of the prediction accuracy. Classical AUC and accuracy measures are problematic in the ambiguous target setup. We focus here on A and B HLA alleles, and not on C. We thus have for each TCR 2 A and 2 B candidate HLA alleles. One can show that in such a case, the maximal AUC that can be achieved is limited by approximately 0.625 (see Appendix A.1 for mathematical explanation).

We propose a new accuracy measure—the fraction of samples for which the model predicts the highest score for a positive pair (further defined as GA). For example, assume that each TCR is associated with 4 candidate HLA alleles and not associated with 60 other HLA alleles absent from the appropriate host. We assign a score to each HLA allele and define the accuracy to be the fraction of TCRs, where the highest score is for one of the 4 out of 64 alleles associated with this TCR.

**Figure 5:**
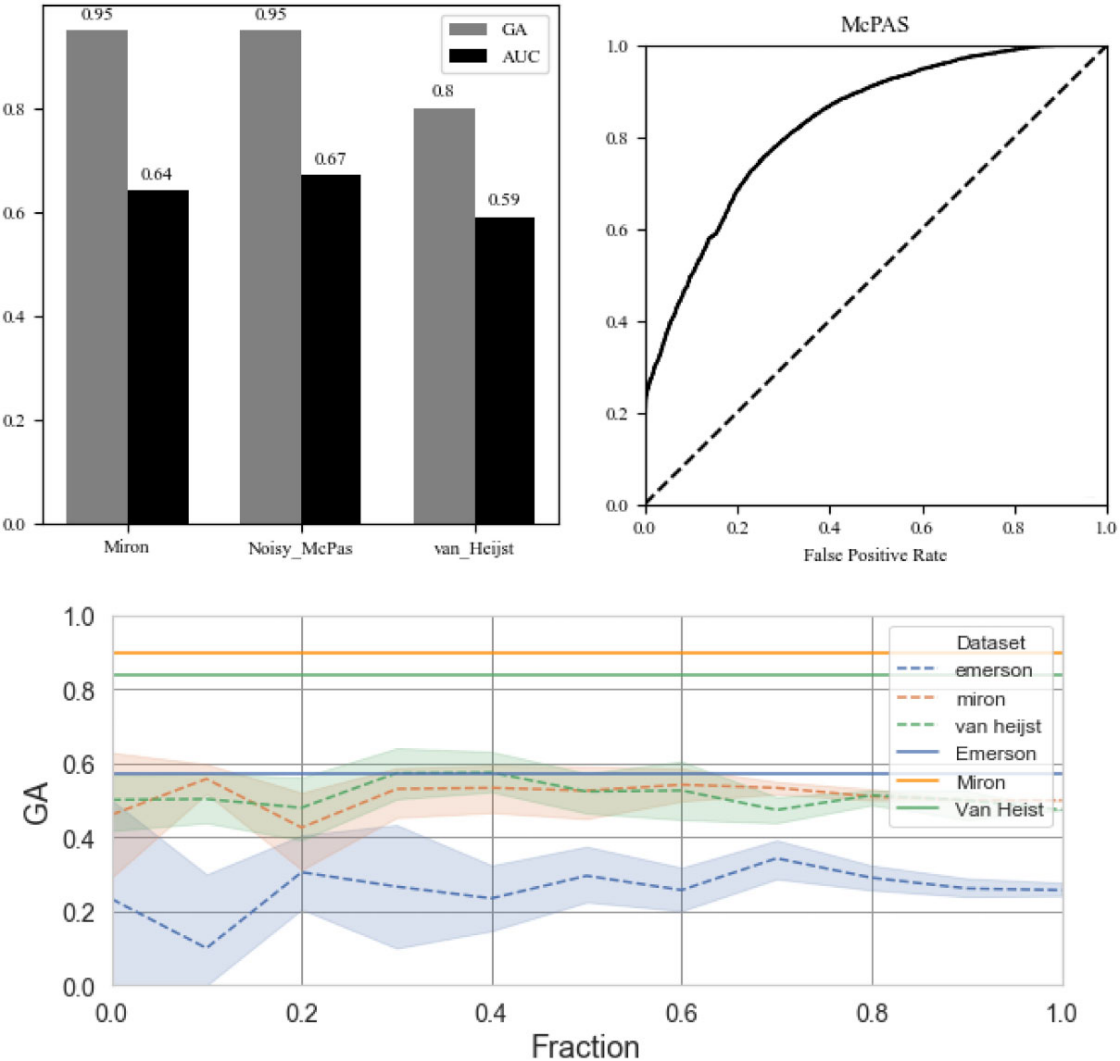
The bar plot in the upper right shows the results of CLAIRE (both GA and AUC) on different datasets. The upper left plot shows here the ROC curve of the model we trained on McPAS dataset, then tested on the original McPAS, and not on the simulated McPAS as explained in ‘Limitations of PTH models in BSE Data’ section. The lower bar plot shows the GA score results for the random scenario, compared to the GA that we get with CLAIRE. The dashed lines are the average GA results over 10 realization per sample size. The sample sizes are represented as a fraction of the total sample used for the analysis. The random realizations are the GA expected when the class of each sample is scrambled. The full regions around the dashed lines are the standard error. The full lines are the real GA obtained for the appropriate dataset.

To compare the GA with its expected value, we scrambled the labels, and computed the expected GA, as a function of the sample size (dashed line with confidence interval in Fig. 5). The confidence interval is computed over 10 different realization of the ML on random samples. The sample size for each dataset is a fraction of the size of the dataset used. One can clearly see that at the sample sizes used for the current analysis (Fraction of 1), the GA has a very narrow distribution. We compared the GA we obtained for each model (full lines with the same colors in the same figure). In all datasets, the real value is far from the random GA (more than 10 standard deviations). Given the robustness of the GA estimate, we use it in the following comparisons in parallel with the AUC.

To test the AUC and GA in a controlled BSE setup, we used the McPAS data and produced a simulated BSE by assigning each TCR with three more HLA alleles using the HLA allele distribution in McPAS. We first tested the GA of the model trained on McPAS and tested on noisy McPAS. We then trained the same model on the noisy McPAS (see ‘Methods’ section for details).

When we trained the model on the real McPAS dataset and tested it on the noisy McPAS, we got a test set AUC of 0.63. When we trained the model on the noisy McPAS and tested it on the noisy McPAS, the test set AUC was 0.66 (i.e. in both trained models the AUC is very close to the maximum AUC that can be achieved on noisy McPAS, but directly training on the noisy McPAS is better). The GA on the test set in both models was very high and similar and was 0.95.

To test the results on real BSE datasets, we used two datasets separated into CD4 and CD8 T cells that further denoted the Miron and van Heijst datasets (Table 3). The first is a memory dataset and the other one is a whole blood dataset. The McPAS dataset is composed of TCRs reported to bind targets, and as such are target specific. The AUC and GA for McPAS are 0.66 and 0.95, respectively. For the Miron dataset, which is a memory dataset, the AUC and GA are 0.58 and 0.9, respectively. However, the van Heijst dataset is not a memory dataset and for this dataset the AUC and the GA are 0.52 and 0.6, respectively, suggesting that target-specific TCRs are the most associated with the HLA, followed by memory cells, and whole blood T cells are the least associated.

**Table 3:** Results of CLAIRE on test sets

Train Test	McPAS	Miron	van Heijst	Emerson
McPAS	GA: 0.95, AUC: 0.66	GA: 0.95, AUC: 0.64	GA: 0.8, AUC: 0.59	Random
Miron	GA: 0.9, AUC: 0.58	GA: 0.9, AUC: 0.58	GA: 0.85, AUC: 0.58	Random
van Heijst	GA: 0.6, AUC: 0.52	GA: 0.6, AUC: 0.52	GA: 0.84, AUC: 0.6	Random
Emerson	Random	Random	Random	GA: 0.57, AUC: 0.58

GA is the new accuracy measure we proposed, as explained in ‘Limitations of PTH models in BSE Data’ section. We trained CLAIRE on different datasets, and we tested each one of the trained models on each one of the datasets. In the first row are the datasets we trained the model on, and in the left column are the datasets that we tested the model on.

### Two-stage CLAIRE for BSE

While the GA and AUC of CLAIRE trained on PTH and tested on the Miron and Van Heijst BSE datasets are much higher than expected randomly, they are still lower than when tested on the noisy McPAS dataset. The simplest explanation would be the affinity difference between TCR experimentally tested to bind a peptide in a context of a given MHC and TCR that passed positive selection in the thymus. We thus propose to directly train a TCR–MHC-binding prediction on BSE datasets. However, classical ML methods expect one class per target, while here each TCR is associated with a subset of possible HLA alleles. To address that, we developed a two-stage learning method, where first all candidate HLA alleles are considered legitimate for the appropriate TCR. Then, at the second stage, only the HLA allele with the highest score in the first stage is considered legitimate (see ‘Methods’ section for details), and a second round of model training is performed only for the top score peptide.

We trained CLAIRE on all datasets and tested it on all the other datasets (see ‘Methods’ section for experimental details). We computed for each the AUC and the GA (Table 3—columns: training and rows: test; Table 4). As mentioned, when CLAIRE was trained on McPAS dataset, the performance was very high on the McPAS test set, but also on the Miron dataset. However, the McPAS dataset does not generalize to the van Heijst dataset. The performance of CLAIRE when trained on the Miron memory cells dataset is also good and is equivalent to the McPAS dataset.

**Table 4:** Test set results when the model is trained on all datasets combined, except for the Emerson dataset

	McPAS	van Heijst	Miron
GA	0.95	0.84	0.85
AUC	0.63	0.53	0.58

GA is the new accuracy we proposed as explained in ‘Limitations of PTH models in BSE data’ section. When we train the model on all the datasets together, the AUC is lower than for each dataset.

The van Heijst whole blood dataset has the lowest performance among the CD8 T cell datasets. It does generalize slightly to the McPAS and Miron datasets, but with very low GA and AUC. Finally, the Emerson dataset composed of both CD4 and CD8 T cells was the worse and did not generalize to any other dataset, and the other datasets do not generalize the Emerson dataset. This last result is expected since a large fraction of the TCRs in the Emerson dataset are actually from CD4 T cells. The McPAS dataset and the Miron dataset both contain memory T cells exclusively, whereas Emerson and van Heijst do not.

One can see a clear order, TCRs known to interact with specific HLA alleles (McPAS) have the highest scores, followed by memory T Cells (Miron), by whole blood cells sorted to be CD8 (van Heijst) and followed by whole blood in general (Emerson). Such an order is precisely the one expected from the binding affinity level required in each dataset. When we learned on any of the CD8 datasets, and applied the model to the Emerson data, the AUC and GA were random. The simplest explanation is that in the Emerson dataset, the TCRs from CD4 T cells are not associated with any Class I HLA. To solve that, one first needs to separate TCRs into those originating from CD4 and CD8 T cells, before we can predict binding to Class I MHC alleles.

### BSE cross validation

As an independent cross validation in the first step of the two-step BSE analyses, we identified TCRs shared by multiple donors (at least T1), and computed for each such TCR the number of time it appeared in a donor with a given HLA. This frequency was normalized by the total HLA probability in the population for each HLA. The resulting ratio was then further normalized to have a sum of 1. TCRs with a normalized ratio of above *r*1 for their most probable HLA were defined to be associated with it. We then computed the CLAIRE prediction for this TCR over all HLA and computed for each HLA, and for each TCR associated with this HLA, whether CLAIR gives the highest score to the associated HLA.

**Figure 6:**
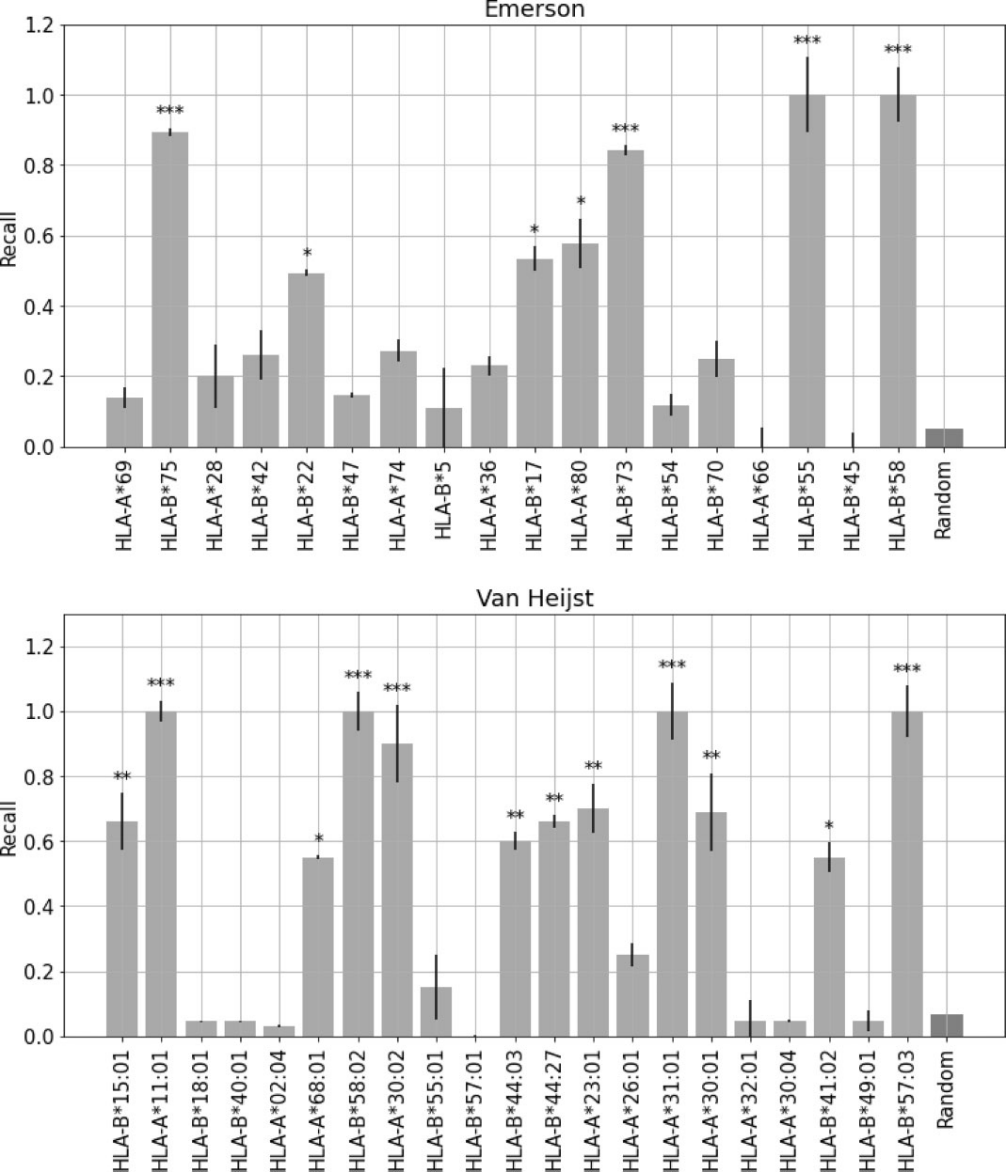
Comparison of CLAIRE results and a random model on TCRs tightly associated with the presence of specific HLA. The right bar represents the Recall of a random model, and the other bars represent the recall of each HLA. For each HLA, we took all the TCR that is associated with that HLA, and calculated how many TCRs got the highest probability to bind to that HLA. The asterisks represent the significance level of a Chi-square test between the results of a random model and CLAIRE. ***P <0.001, **P<0.01, *P<0.05. The upper plot is for the Emerson data. The lower plot is for the van Heijst.

We ran that experiment on two datasets: Emerson and van Heijst. In the Emerson experiment, we chose T1=15,r1=0.5 and in the van Heijst data, T1=5,r1=0.25. We then compared between the results of CLAIRE and a random model on the TCRs from both of the experiments, and we saw that CLAIRE predicted higher probability for binding the ‘right’ than the random model. We performed a chi-squared test to determine whether there is a statistically significant difference between the results of a random model and CLAIRE, and we marked with asterisks the bars of the HLAs with a high significance level (see Fig. 6).

### CD4–CD8 classifier

We thus built a binary classifier that predicts whether a T cell is a CD8 T cell (1) or a CD4 T cell (0). The TCR representation of the CD4–CD8 T cell classifier is similar to the one of the TCR**–**HLA classifier, as well as the encodings. The output is 1 if it is a CD8 T cell and 0 if it is a CD4 T cell (see Methods for model architecture in ‘CD4-CD8 classification neural network’ section). We used the McPAS, VDJdb and Sallusto dataset that contain both CD4 and CD8 T cells from 49 different samples. We also used the van Heijst dataset that contains CD4 and CD8 T cells from 27 patients, the Gold dataset that contains CD4 and CD8 T cell repertoire longitudinally throughout pregnancy, and finally the Miron dataset, that contains CD4 and CD8 memory T cells (see Table 1).

**Figure 7:**
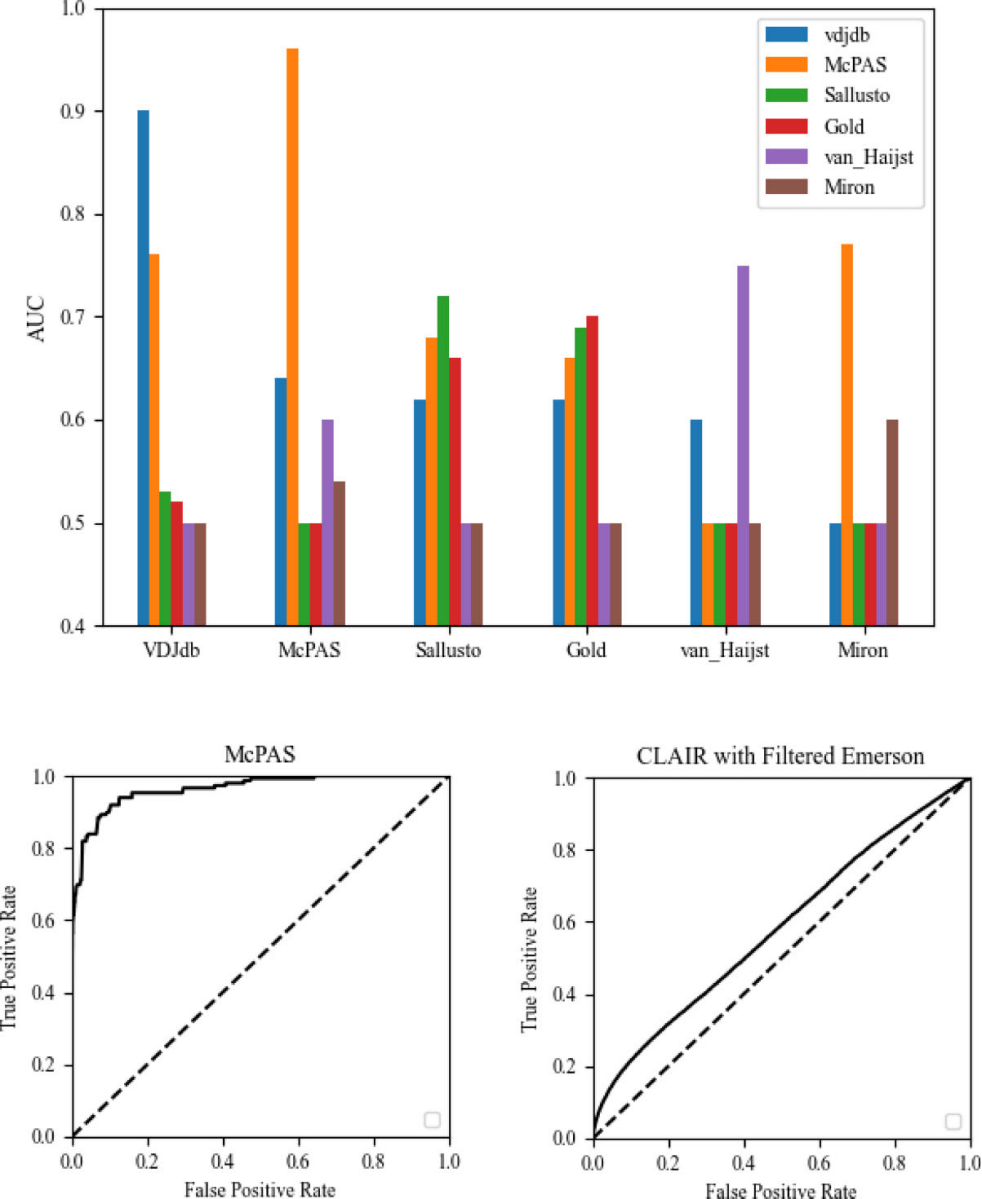
The bar plot represents the AUC score of the CD4–CD8 model. The *x*-axis is the datasets we train the model on, and each color represents the datasets we tested the model on. The *y*-axis is the AUC score. This plot represents the results as in Table 5. The bottom left plot is the ROC curve of the CD4–CD8 model when we train it on McPAS dataset. The bottom right plot is the performance of CLAIRE on the Emerson dataset using only the T cells that are most likely to be CD8 T cells (top 0.01%), using the CD4–CD8 model.

We again divided each dataset to training and test sets, and performed internal validation within the same dataset, or cross-dataset validations, where the model is trained on a dataset and tested on another dataset (see Fig. 7 and ‘CD4–CD8 classification neural network’ section for details).

The highest performance was obtained when training the model on VDJdb and testing it on VDJdb (Tables 5 and 6). VDJdb generalized to McPAS, but did not generalize to any other dataset. When we trained the model on the McPAS dataset, it had a high performance on itself. It generalized well to VDJdB and to the van Heijst. The Sallusto dataset did not generalize any other dataset except Gold, but with low accuracy. When we trained and tested the model on the Sallusto dataset the accuracy was fine. The Gold dataset generalized to all the datasets, except from the van Heijst and Miron dataset. Van Heijst dataset does not generalize well to any of the other datasets, and has fine accuracy when we test the trained model on van Heijst.

**Table 5:** CD4–CD8 model AUC scores on test sets when trained on different datasets

Train Test	VDJdb	McPAS	Sallusto	Gold	van Heijst	Miron
VDJdb	0.9	0.64	0.62	0.62	0.6	0.5
McPAS	0.76	0.96	0.68	0.66	0.5	0.77
Sallusto	0.53	0.5	0.72	0.69	0.5	0.5
Gold	0.52	0.5	0.66	0.7	0.5	0.5
van Heijst	0.5	0.6	0.5	0.5	0.75	0.5
Miron	0.5	0.54	0.5	0.5	0.5	0.6

We trained six different models with CD4–CD8 labels, to predict if a T cell is a CD4 T cell or a CD8 T cell. We tested all the trained models on each one of the datasets. Details on each one of the datasets are in ‘Datasets’ section.

**Table 6:** CD4–CD8 model GA scores on test sets when trained on different datasets

Train Test	VDJdb	McPAS	Sallusto	Gold	van Heijst	Miron
VDJdb	0.94	0.84	0.13	0.7	0.4	0.1
McPAS	0.91	0.91	0.14	0.64	0.4	0.74
Sallusto	0.25	0.4	0.78	0.55	0.9	0.7
Gold	0.54	0.5	0.45	0.7	0.6	0.5
van-Heijst	0.5	0.9	0.5	0.5	0.7	0.6
Miron	0.2	0.3	0.7	0.2	0.7	0.57

This table is similar to Table 5, but with the accuracy score.

The Miron dataset generalizes only to the McPAS dataset, and has low performance on itself. So, it seems that while memory T cells BSE are highly useful for classifying HLA, they are not appropriate for classifying CD4 and CD8 T cells. We also trained for each dataset all the other datasets combined, and tested the model on the dataset we left out of training. The results are in Table 7.

**Table 7:** CD4–CD8 model AUC and GA scores on test sets when trained on combined datasets

	McPAS	VDJdb	Sallusto	Gold	van Heijst	Miron
GA	0.9	0.61	0.54	0.6	0.69	0.2
AUC	0.93	0.62	0.55	0.63	0.73	0.53

In order to predict if a T cell is CD4 or CD8, we trained all the six datasets together, and tested the trained model of each one of the datasets. The test set accuracy of this model is in the first row, and the AUC is in the second row. Each one of these datasets has CD4–CD8 labels, as explained in ‘Datasets’ section.

We used the CD4–CD8 model to limit the TCR**–**HLA prediction in the Emerson dataset to TCRs with the highest probability of originating from CD8 T cells (top 5% scores of the CD4–CD8 Classifier). For those, CLAIRE was able to predict the HLA from the TCR in the Emerson dataset (Table 3). Yet, the Emerson dataset did not generalize to other datasets, and other datasets did not generalize to the Emerson dataset, even when only TCRs predicted to originate from CD8 T cells were used. The failure of the transfer learning suggests that the selected subset of T cells is not representative.

## DISCUSSION

Within the TCR*β* chain, the CDR1 and CDR2 of the TCR contact the MHC α-helices, and affect TCR**–**MHC binding [1]. We confirmed the correlation between the CDR1 and CDR2 regions of a TCR and its HLA association, using a Naive Bayes classifier (‘Naive Bayes V*β* gene-based MHC-binding prediction’ section). Including the information about the CDR3 region improves the prediction. Using the CLAIRE prediction algorithm on the labeled McPAS dataset, we produced a high accuracy predictor of MHC–TCR binding. To the best of our knowledge, CLAIRE is currently the first TCR–MHC-binding prediction algorithm. The effect of the CDR3 can be direct or indirect via the bound peptide. An important limitation of the predictor trained on the McPAS dataset is that it did not transfer well to BSE datasets. We thus developed a two-stage predictor, where each TCR is assigned a group of candidate HLA. We labeled all the TCRs in a subject’s repertoire as positive for all of four HLA-A and HLA-B alleles, although in reality most TCRs were probably selected on a single one of them. Thus for each correct (TCR and HLA) positive pair, we entered three false-positive pairs. Then we chose for each TCR only the pair that got the highest prediction score out of the 4, and performed a second round of training, when a single HLA was assigned to each TCR. Despite the noisiness of the training and test sets, this novel two-stage approach led to high accuracies. We defined beyond the AUC a novel accuracy that measured appropriate for such ambiguous cases—the GA. Note that additional iterations of the same process may further improve its accuracy.

In order to prove that the pairings made by CLAIRE after the first run of the noisy dataset are actually a good approximation of real-world TCR**–**HLA pairs, we predicted the HLA from the TCR on a noisy version of the McPAS dataset that has real-world HLA labeling for each TCR. First, we added noise to the TCR**–**HLA pairs, by adding three wrong TCR**–**HLA pairs for each TCR. The AUC score when training on the noisy dataset was actually higher than when training on the clean dataset. We then further trained and tested CLAIRE on BSE datasets. The CLAIRE predictor obtained a high accuracy on all CD8 memory datasets, and a lower accuracy on whole blood CD8 dataset. This consistent difference suggests that memory T cells may be more associated with the host HLA than naive cells.

We then further tested CLAIRE to datasets containing both CD4 and CD8 T cells. The Emerson dataset [41] contains immune repertoires of subjects, and their HLA-A and HLA-B alleles. To classify TCRs from such cells, we developed a CD4–CD8 classifier to filter the TCRs that belong to MHC Class II out of the repertoire.

In the process of predicting bindings on BSE datasets, some assumptions and simplifications were made. In the case of mixed CD4–CD8 T cells, some TCRs that belong to Class II may have been included in the filtered dataset. Moreover, we ignored the C locus as many of our predictions are wrong per definition. We ignored HLA-C, since it is lacking for many datasets.

Transferring conclusions made on the McPAS dataset to BSE datasets may be problematic. Those datasets have different distributions of both TCR and HLA and represent different experimental setting. Moreover, generating three false pairs for each TCR on the McPAS dataset is not exactly tantamount to creating four pairs for each TCR in a repertoire, since there are differences in the HLA distributions.

Our results are consistent with previous results showing that CDR3 loops determine the connection between the antigen-peptides and the TCR and that MHC-I alleles shape the CDR3 repertoire [65]. However, we have here extended these results to be able to predict the effect of the HLA on TCR and predict TCR**–**MHC binding. Moreover, we suggest a difference between memory and naive cells in the effect of HLA on the TCR, and propose that further efforts to correlate TCR and HLA should be focused on memory cells.

## Figures and Tables

**Figure A1:**
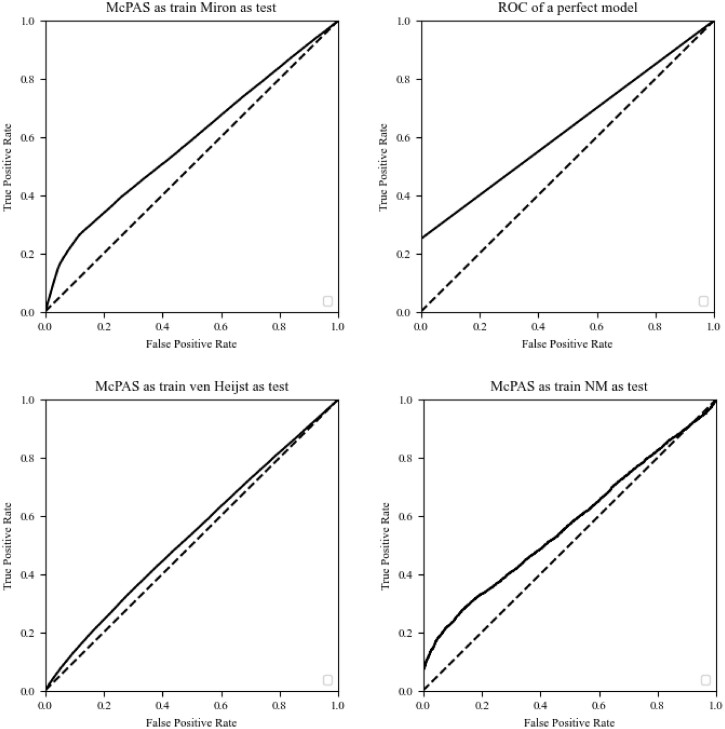
Results of CLAIRE. We show here a ROC curve of a perfect model under the conditions of CLAIRE, as explained in ‘Limitations of PTH models in BSE Data’ section. Next to the perfect ROC curve we demonstrate the ROC curves of CLAIRE when trained on different datasets. The first dataset is McPAS, contains binding pairs of a memory T cells, and a *HLA* gene. The second dataset is Miron dataset, contains memory T cells from 11 donors, 9 of them with HLA typing. The last one is van Heijst dataset, contains TCRs from 27 patients with HLA typing.

## APPENDIX A

### Appendix A.1 Mathematical explanation for bound AUC

When attempting to use a PTH model on a BSE data, as described in ‘Limitations of PTH models in BSE Data’ section, the maximal AUC that can be achieved in such a case is limited by approximately 0.625. The reason for that is, that in the ‘True-Positive Rate’ axis, the maximum value is 0.25—the ratio the positive samples that the model predicted as positive and the positive samples. From the (0, 0.25) point, the graph continues linearly to (1, 1), since the model separation between the negative and the positive samples that are most likely to be negative, is random (Fig. 5). Note that if the distribution of TCR association with different alleles is not random, this number can be slightly higher, and if many TCRs are associated with C alleles that are not included here, the number can be lower.

**Table A1:** A list of notable acronyms in the paper and their long-form meaning

Acronym	Expansion
AUC	Area under curve
TCR	T cell receptor
PTH	Paired TCR–HLA
BSE	Bulk sequencing experiments
MHC	Major histocompatibility complex
HLA	Human leukocyte antigen
AA	Amino acid
GA	Group accuracy
